# Chest wall stabilization following hemi-sternectomy assisted by 3D printed model

**DOI:** 10.1080/23320885.2026.2627738

**Published:** 2026-02-11

**Authors:** Phillip L. Nichols, Caleb W. Brown, Derek C. Wenger, Jennifer M. Osher, Jeremy M. Powers

**Affiliations:** ^a^ Quillen College of Medicine, East Tennessee State University, Johnson City, TN, USA; ^b^ Department of Surgery, Division of Plastic and Reconstructive Surgery, Quillen College of Medicine, East Tennessee State University, Johnson City, TN, USA

**Keywords:** Sternal reconstruction, sternectomy, hemi-sternectomy, 3D printing, reconstruction

## Abstract

The incidence of deep sternal wound infection post-sternotomy is 1.6%. Treatment with sternectomy or hemi-sternectomy can result in exposed thoracic organs, chest wall and shoulder girdle instability, reduced upper extremity function and disability. Various materials and soft tissue techniques are available for chest wall reconstruction; however, the use of a 3D-printed model to facilitate preoperative bending of titanium plating systems for sternal reconstruction has not been previously reported. A 51-year-old female presented to the plastic surgery clinic with a history of coronary artery bypass grafting complicated by sternal osteomyelitis. A left hemi-sternectomy was performed to treat the infection, after which the patient developed chronic pain and shoulder instability. The patient was referred to plastic surgery to consider options for combined sternal stabilization and flap coverage. A polyamide 3D-printed model of the sternum and ribcage was produced, followed by pre-operative bending of sternal-spanning long rib plates for use in the operating room. The model and the pre-bent plates were sterilized and brought to surgery. After elevation of pectoralis muscle flaps and exposure, the pre-bent plates were directly applied to the rib cage with minimal modification, thus obtaining the pre-planned reduction of the sternum/ribcage and saving a significant amount of time in the operating room. The patient made a good recovery from the operation with significant improvement of chest wall stability, improvement of shoulder function, and a decrease in chest discomfort. This report details the application of 3D printing to increase the precision and efficiency of chest wall stabilization.

## Introduction

The incidence of deep sternal wound infection following cardiothoracic operation is 1.6% [1,2]. Sternectomy and hemi-sternectomy can result in exposed thoracic organs, chest wall and shoulder girdle instability, reduced upper extremity function, and disability [3,4]. Various materials and soft tissue techniques are available for chest wall reconstruction; however, current literature lacks comparative studies. In postoperative osteomyelitis of the sternum in which substantial bone removal is required, traditional sternal reconstruction methods may prove insufficient due to the inability to span areas of missing bone. Addressing this challenge involves utilizing plates designed to anchor onto the remaining bone or the adjacent rib segments. Accounting for the rib contour intra-operatively requires plate bending and taking the hardware in and out of the surgical field many times, often taking up a prolonged portion of the case. We present a clinical case of a sternal-spanning fixation system which was pre-bent to a 3D-printed model prior to surgery, saving significant time in the operating room.

## Patient

A 51-year-old female presented to the plastic surgery clinic with a past medical history of hypertension, diabetes, morbid obesity, and coronary artery disease status post coronary artery bypass grafting (CABG). At six months post-op, this was complicated by deep sternal wound infection/sternal osteomyelitis. A left hemi-sternectomy was performed by the cardiac surgeon with removal of sternal wires and primary skin closure. The patient developed chronic pain and left shoulder instability that was not responsive to physical therapy. The patient desired improvement in her ability to function as well as pain relief.

When considering her surgical options, several unique challenges arose. The absence of the left hemi-sternum made it difficult or impossible to use standard sternal fixation plates. The midline nature of the defect and the possible future need to re-enter the chest made it inadvisable to use long rib plates to span across the sternal defect. Pin-adjoined sternal/rib plates were not felt to provide enough stability across the bony defect. After comparing available options, the Talon Recon system (KLS Martin, Jacksonville, FL) was chosen.

**Figure 1.**
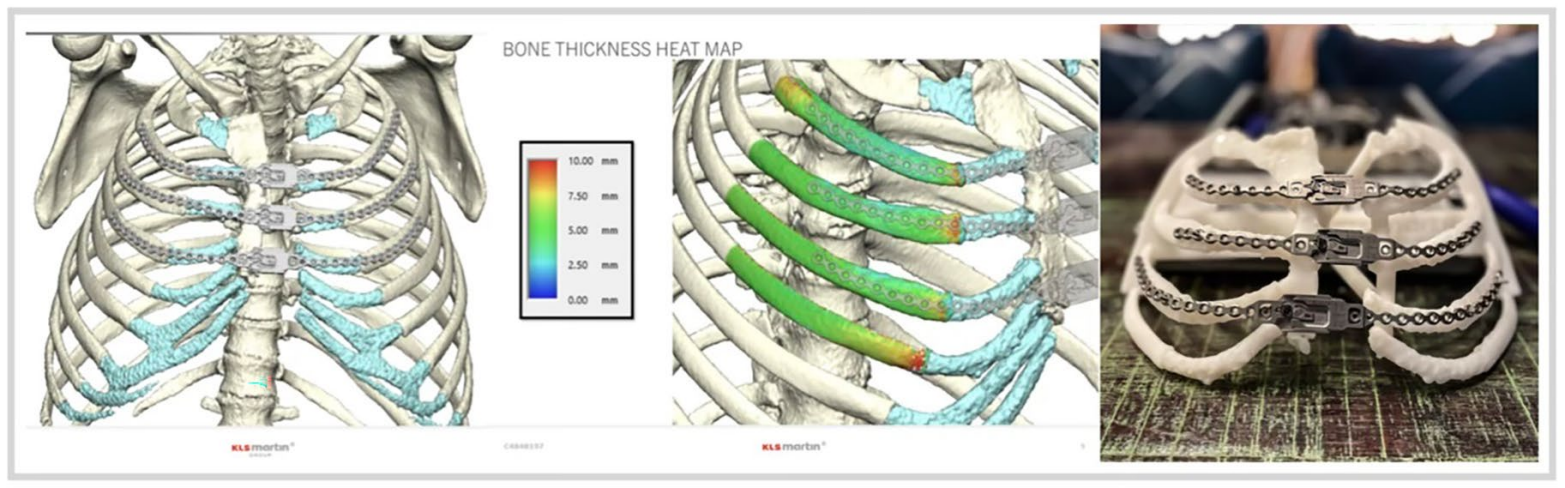
3D renderings of the patient’s remaining sternum and rib anatomy with predicted location of Talon Recon plates (left). Bone thickness heat map to assist in determining screw length (middle). 3D printed polyamide model of the chest wall with Talon Recon plates pre-bent prior to surgery (right).

Prior to surgery, thin cut CT Chest was obtained, and surgical planning was utilized to perform a ‘virtual bony reduction.’ KLS Martin then produced a polyamide 3D-printed model of the reduced sternum and ribcage using stereolithography. The model was used as a template on which to bend the Talon Recon plates prior to surgery (Figure 1). Three plates were pre-bent to the left and right segments of the second, third, and fourth ribs using the model, and excess holes at the ends of each plate were trimmed. Plates were then sterilized at the hospital and brought to the operating room for implantation during surgery.

**Figure 2.**
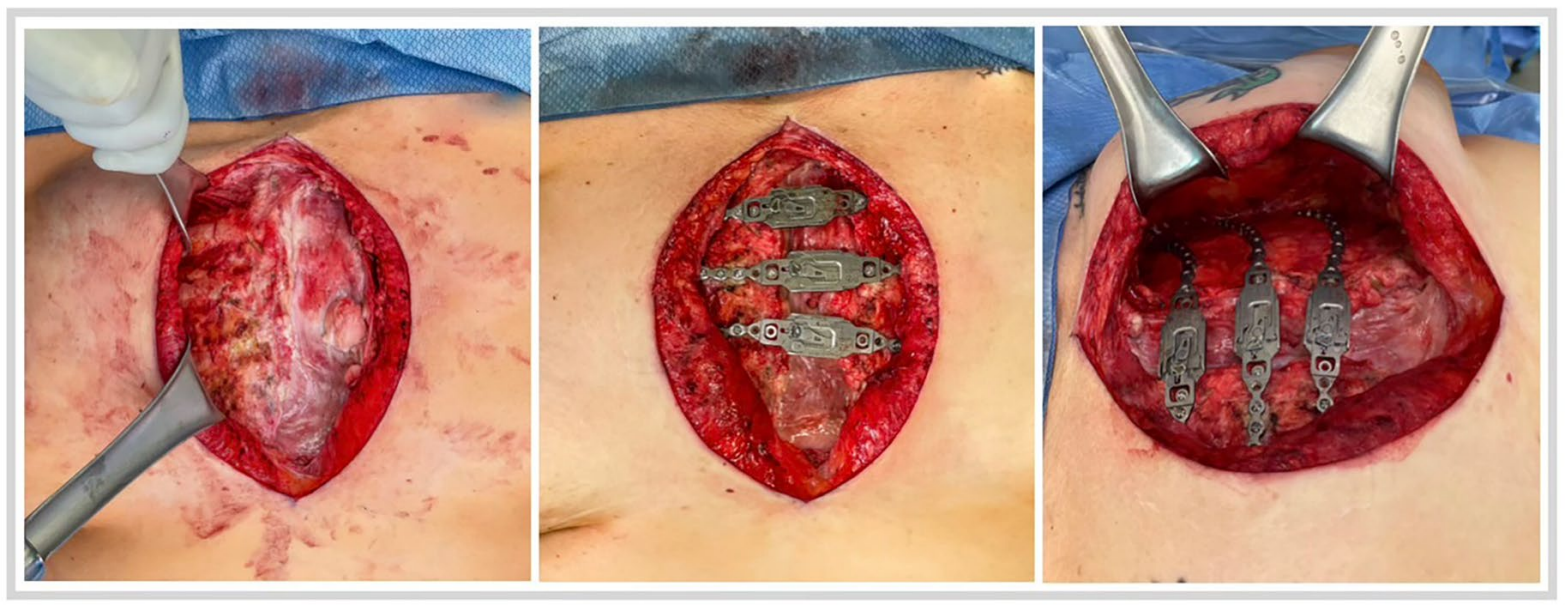
Re-opening of midline sternal scar with absence of left half of sternum (left). KLS Martin Talon Recon plates in position on left and right second, third, and fourth ribs, spanning the sternal defect (middle and right). Identifying marks have been obscured.

In the operating room, the midline sternotomy scar was opened, and bilateral pectoralis muscle flaps were elevated from the chest wall and ribcage. The pre-bent Talon Recon plates were placed and secured with self-drilling screws whose depth was estimated based on the bony thickness ‘heat map’ (Figure 2). The pectoralis muscle flaps were advanced to the midline and closed to each other over drains with multiple inverted figure-of-eight sutures.

## Results

The patient tolerated the procedure well with no immediate complications. On postoperative day one, the patient was found to have bilateral pneumothoraces on routine chest X-ray, which resolved upon chest tube management. The patient was ultimately discharged on postoperative day seven. She was seen three months postoperatively with much improved stability of the chest wall, improved shoulder function, and less pain with a continued physical therapy regimen.

Fifteen and a half months following her index operation, the patient underwent a hardware revision due to the development of chest pain resulting from hardware failure at the fourth rib. Intraoperatively, the second rib hardware on the right side was also noted to be slightly loose.

During the operation, the same 3D model from her index operation the year prior was used to facilitate re-bending of hardware at the second rib, replacement of hardware at the fourth rib, and the addition of a bridging plate at the lower sternal boarder that contoured to the fifth rib on both sides. Evaluation in clinic on postoperative day thirteen demonstrated appropriate wound healing with chest pain improvement. Further follow ups have demonstrated maintenance of chest wall stability and resolution of symptoms.

## Discussion

Three-dimensional (3D) technologies have increasingly influenced thoracic reconstruction over the past two decades, with applications spanning simple chest wall deformities, complex congenital malformations, and extensive defects following oncologic resection. The introduction of 3D virtual reconstruction and patient-specific implant planning enabled preoperative determination of implant length, orientation, and curvature, reducing the need for intraoperative bending maneuvers and minimizing bar-related complications [5]. Additional applications of this approach include the development of 3D-printed templates and real-size chest wall models, which have facilitated implant customization, operative simulation, and patient education, ultimately shortening operative times and improving anatomic conformity and surgical outcomes [5]. Building on this foundation, 3D technology has also been adopted for the correction of more complex chest wall deformities, where 3D printing of cutting templates, surgical guides, and anatomical ribcage models has enabled precise osteotomies, implant verification, and multimodal preoperative planning with high procedural accuracy and minimal intraoperative revision [5].

Furthermore, 3D-assisted reconstruction has expanded substantially in oncologic surgery, where full- or partial-thickness thoracic wall resections demand solutions that restore physiologic motion, protect intrathoracic structures, and re-establish skeletal support. In this domain, 3D modeling allows surgeons to simulate resections, design patient-specific implants, and manufacture prostheses in titanium, polyetheretherketone (PEEK), and methyl methacrylate, improving implant fit, decreasing operative time, and enhancing functional outcomes [5–7]. These advances collectively demonstrate the versatility and clinical value of 3D technologies across the thoracic reconstruction spectrum—from implant planning and contouring to full prosthesis generation—and highlight their ability to reduce operative complexity, optimize implant geometry, and improve patient-centered outcomes. In addition, 3D-printed models have been shown to enhance preoperative visualization and operative decision-making by providing surgeons with accurate, reproducible representations of thoracic anatomy, improving assessments of resectability and surgical approach beyond conventional imaging alone [8].

In the present case, the incorporation of 3D technology proved integral to successful reconstruction, functioning in a manner analogous to its established role in post-oncologic chest wall surgery, where sizable osseous defects require precise preoperative planning. Following hemi-sternectomy, the patient’s residual sternal defect created a complex anatomic landscape that would have been difficult to address using conventional intraoperative contouring alone. The 3D-printed thoracic model enabled accurate virtual reduction of the ribcage and facilitated pre-bending of the fixation plates to the patient’s specific anatomy, allowing the implants to be applied directly without substantial modification. This approach not only reproduced the pre-planned reduction with high fidelity but also reduced operative time by eliminating iterative plate removal and recontouring. Furthermore, minimizing implant manipulation within the surgical field may reduce contamination and subsequent infection risk—complications well documented in titanium rib bridge systems—along with pneumothorax, hematoma, and the less common occurrences of dislocation and hardware failure [9]. These advantages underscore the broader premise that thoughtful integration of 3D modeling, strategic implant selection, stable soft tissue coverage, and meticulous postoperative care are essential components in optimizing outcomes for patients requiring complex chest wall stabilization.

In addition to the advantages conferred by preoperative 3D modeling, the choice of fixation hardware was central to the success of this reconstruction. The KLS Martin Talon Recon system was selected because, unlike traditional plating constructs that rely on direct sternal purchase, it enables secure stabilization across the midline by anchoring to the ribs, the residual sternum, or both [10]. This capability was particularly relevant in the present case, where hemi-sternectomy eliminated sufficient sternal bone stock to support conventional sternal plates. The design of the Talon Recon system allowed the construct to span the defect effectively without requiring fixation to compromised sternal segments, thereby restoring structural stability while respecting the altered anatomy. Moreover, a defining feature of this system is its capacity for controlled re-entry into the thoracic cavity, an advantage of substantial importance for patients with prior coronary artery bypass grafting who may require future cardiac interventions. By providing rigid stabilization without sacrificing the ability to reopen the construct when necessary, the Talon Recon system addressed both the immediate biomechanical demands of reconstruction and the long-term clinical considerations unique to this patient.

The value of the present case lies in the combined use of a patient-specific 3D-printed thoracic model with the KLS Martin Talon Recon system for sternal reconstruction after hemi-sternectomy. While 3D models can facilitate precise preoperative planning and implant contouring, and the Talon Recon system independently provides rigid fixation with the option for controlled re-entry into the thoracic cavity, their purposeful integration allowed us to create a tailored construct that restored chest wall stability without complicating future mediastinal access in the context of altered surgical anatomy.

## Conclusion

Virtual surgical planning can be utilized preoperatively to increase the precision of sternal reconstruction, decrease patient time under anesthesia, and may decrease risk of contamination and infection. Using 3D-printed models to pre-bend plates can be effectively implemented for sternal reconstruction, creating a stable construct that is precisely suited to individual patients’ needs. In addition to immediate benefits in the operating room, utilizing a device that includes a quick-release mechanism ensures additional long-term benefit of easier access to the mediastinum in the event of future cardiothoracic surgery. This is of particular importance due to this patient’s relatively young age, comorbidities, and potential need for future coronary intervention. Longer follow-up and reports on other cases utilizing this system are required to confirm the generalizability of our findings in improving patient outcomes, and literature lacks comparative studies with other techniques.

## Data Availability

The data that support the findings of this case report are not publicly available due to privacy restrictions.
